# Modelling diseases with relapse and nonlinear incidence of infection: a multi-group epidemic model

**DOI:** 10.1080/17513758.2014.912682

**Published:** 2014-04-29

**Authors:** Jinliang Wang, Jingmei Pang, Xianning Liu

**Affiliations:** ^a^ School of Mathematics and Statistics, Southwest University, Chongqing400715, People's Republic of China; ^b^ School of Mathematical Science, Heilongjiang University, Harin150080, People's Republic of China

**Keywords:** global stability, relapse distribution, multi-group epidemic model, Lyapunov functional, 34D30, 92D30

## Abstract

In this paper, we introduce a basic reproduction number for a multi-group SIR model with general relapse distribution and nonlinear incidence rate. We find that basic reproduction number plays the role of a key threshold in establishing the global dynamics of the model. By means of appropriate Lyapunov functionals, a subtle grouping technique in estimating the derivatives of Lyapunov functionals guided by graph-theoretical approach and LaSalle invariance principle, it is proven that if it is less than or equal to one, the disease-free equilibrium is globally stable and the disease dies out; whereas if it is larger than one, some sufficient condition is obtained in ensuring that there is a unique endemic equilibrium which is globally stable and thus the disease persists in the population. Furthermore, our results suggest that general relapse distribution are not the reason of sustained oscillations. Biologically, our model might be realistic for sexually transmitted diseases, such as Herpes, Condyloma acuminatum, etc.

## 1. Introduction

In recent years, great attention has been paid in analysing the multi-group epidemic models, which have been proposed to describe the disease transmission dynamics of many infectious diseases in heterogeneous host populations, such as measles, mumps and gonorrhoea and vector-borne diseases such as West-Nile virus and Malaria. For more and detailed justifications for multi-group disease models and many different types of heterogeneity epidemic models (see e.g. [3,13,14,16,20,23,32] and references cited therein). It is well known that long-time behaviours of multi-group models with higher dimensions, especially the global asymptotical stability of the endemic equilibrium (EE), is a very challenging topic. The question of uniqueness and global stability of the EE, when the basic reproduction number is more than one, has largely been open. In [13], a graph-theoretic approach was developed, which is known to be an effective tool for the global stability analysis of multi-group epidemic models. Much research has been done with this approach [3,13,14,22,23,35], focusing on understanding the transmission mechanism and the global behaviour of multi-group epidemic models. So the study of relapse distribution and different nonlinear incidence of infection have been the subject of intense theoretical analysis on the heterogeneous epidemic models in the literature [26,34,35].

In this paper, motivated by the works of van den Driessche and Zou [10], Wang *et al.* [34] and Sun and Shi [31], we shall investigate the global dynamics of a general multi-group epidemic model with general relapse distribution and nonlinear incidence rate, in particular to investigate the impacts of heterogeneity and nonlinear incidence rate on the dynamics of the basic SIR epidemic model. The population is divided into *n* distinct groups (*n*≥1). For 1≤*i*≤*n*, the *i*th group is further partitioned into three compartments:

*S*_*i*_: susceptible individuals in the *i*th group;
*I*_*i*_: infectious individuals in the *i*th group;
*R*_*i*_: recovered individuals in the *i*th group,

and we denote the populations of individuals at time *t* in each compartment by *S*_*i*_(*t*), *I*_*i*_(*t*) and *R*_*i*_(*t*), respectively. Within the *i*th group, let 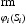 represent the growth rate of *S*_*i*_, which includes both the production and the natural death of susceptible individuals. Typical assumptions on 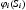 are the following:

(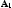) ϕ_*i*_ are *C* ^1^ non-increasing function on [0, ∞) with 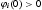, and there is a unique positive solution 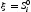 for the equation 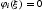. 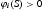 for 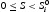, and 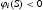 for 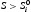, that is,

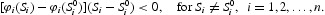

The class of 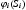 that satisfy (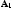) include both 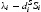 and 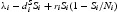, which have been widely used in the literature of population dynamics [1,13].

Since nonlinear incidence of infection has been observed in disease transmission dynamics, it has been suggested that the standard bilinear incidence rate shall be modified into a nonlinear incidence rate in many research [18,28,29]. In this paper, we replace the incidence rate *f*(*S*)*I* in [34] by a general form *f*(*S, I*). We assume that the disease incidence in the *i*th group can be calculated as

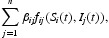

where the sum takes into account cross-infections from all groups, and β_*ij*_ represents the transmission coefficient between compartments *S*_*i*_ and *I*_*j*_. Throughout the paper, β_*ij*_ is non-negative for all 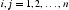, and *n*-square matrix 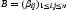 is irreducible [4]. Biologically, this is the same as assuming that any two groups *i* and *j* have a direct or indirect route of transmission. More specifically, individuals in *I*_*j*_ can infect ones in *S*_*i*_ directly or indirectly.

Denote *P*_*i*_(*t*) by the fraction of recovered individuals remaining in the recovered class *t* time units after recovery in each group. It was assumed in [10] that *P*_*i*_(*t*) satisfies the following reasonable properties:

(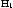) 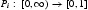 is non-increasing, piecewise continuous with possibly finitely many jumps and satisfies *P*_*i*_(0^+^)=1; 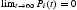 with 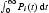 is positive and finite.

The proportion of recovered individuals in the *i*th group can be expressed by the integral

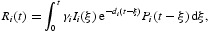

where γ_*i*_>0 is the recovery rate constant assuming that the infective period is exponentially distributed in the *i*th group. The term 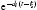 in the above integral accounts for the death of infectives in *i*th group. It is assumed that no individuals are initially in the recovered class, i.e. *R*_*i*_(0)=0. Differentiating *R*_*i*_(*t*) gives

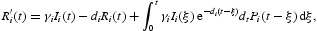

Here, the integral is in the Riemann–Stieltjes sense to allow for possible jump discontinuities of *P*_*i*_(*t*).

**Fig. 1.**
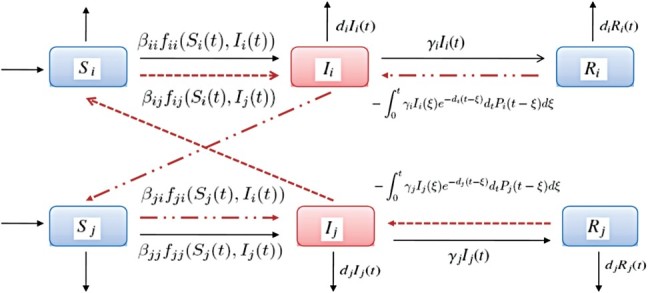
Transfer diagram for model (1).

Hence, the new multi-group epidemic model with relapse distribution and nonlinear incidence rates can be written as the following 3*n*-dimensional system of differential and integral equations:

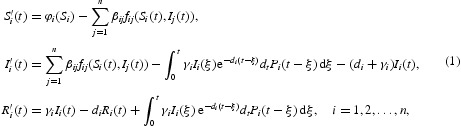

which is an improvement of our earlier model studied in [34]. It also covers many research works in the literature, for example, the ones in [10,26]. The disease transmission diagram is depicted in Figure 1. And the parameters in the model are summarized in the following list:

β_*ij*_: coefficient of transmission between compartments *S*_*i*_ and *I*_*j*_;
d_*i*_: natural death rates of all compartments in the *i*th group;
γ_*i*_: rate of recovery of infectious individuals in the *i*th group.

In the present paper, our goal is to carry out a complete mathematical analysis of system (1) and establish its global dynamics. By virtue of both the relapse distribution and multi-group structure, model (1) is thought to be realistic for sexually transmitted diseases such as herpes simplex virus type 2 (Herpes) [6,7,33], for which disease is transmitted by close physical or sexual contact, recovered individuals may revert back to the infective class because of reactivation of the latent infection or incomplete treatment [7,33]. One important feature of herpes is that an individual once infected remains infected for life, and the virus reactivates regularly with reactivation producing a relapse period of infectiousness (see e.g. [6] and references cited therein). Therefore, the detailed mathematical analysis for model (1) is an important task from not only mathematical but also biological points. It is thus of interest to investigate whether sustained oscillations are the result of general relapse distribution. This provides us with one motivation to conduct our work.

The paper is organized as follows. In the next section, we give some preliminaries on system (1). Our main results are stated in Section 3. One case for *n*=1 is investigated in Section 4. Finally, we give a brief summary and discussion.

## 2. Preliminaries

We first consider system (1) in the phase space ℝ^3*n*^ and with the initial conditions

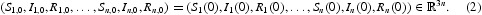

On the basis of biological considerations, we make the following assumptions for 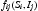 [31]:

(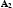): 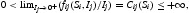 for 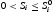.
(**A**_3_) 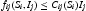 for all *I*_*j*_>0.
(**A**_4_) 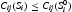, for 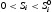, 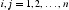.

Typical examples of 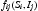 satisfying 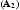
–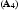 include common incidence functions such as 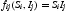 [12,13,19]; 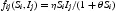 [1,11]; 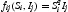 [24,25,37].

lemma 2.1 For initial conditions in Equation (2) with 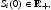 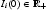 and 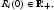 the solutions of system (1) are ultimately uniformly bounded in ℝ^3*n*^.

*Proof* It follows from 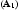 and the first equation of Equation (5) that 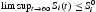 for all *i*=1, 2, … , *n*. Next we show that the solution of system (1) is ultimately bounded in 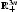. For each *i*, adding the three equations in Equation (1) gives

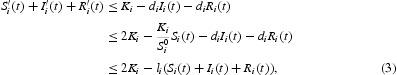

where

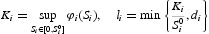

for *i*=1, 2, … , *n*. Hence,

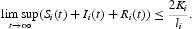

It follows from 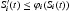 and 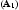 that if 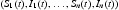 is a solution satisfying 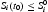 for some *t*_0_>0, then 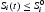 for all *t*≥*t*_0_. By Equation (3), we can also obtain that, for any *i*=1, 2, … , *n*, if 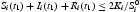 for some *t*_1_>0, then 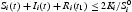 for all *t*≥*t*_1_. Therefore, the set

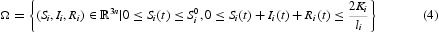

is a forward invariant compact absorbing set with respect to the system (1). Let

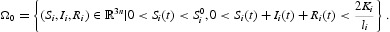

It can be shown that Γ_0_ is the interior of Γ. Furthermore, all positive semi-orbits in Γ are precompact ℝ^3*n*^ [2] and thus have non-empty ω-limit sets.

Note that the first two equations of system (1) are independent from *R*_*i*_, and therefore, the dynamics is governed by the reduced system

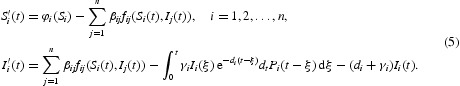

The initial condition of system (5) is assumed to be given as

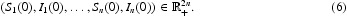

For system (5) with properties (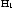), the existence, uniqueness and continuity of solutions follow from the theory for integro-differential equations in [30]. Moreover, it can be verified that every solution of Equation (5) with non-negative initial data remains non-negative. It follows from Equation (4) that the set

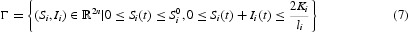

is the subset of Ω. Thus,

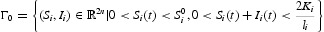

is also the subset of Ω_0_. Furthermore, it is a forward invariant compact absorbing set with respect to the system (5). All positive semi-orbits in Γ_0_ are precompact ℝ^2*n*^ and thus have non-empty ω-limit sets.

System (5) always admits a disease-free equilibrium (DFE), 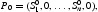 in Γ_0_. Let

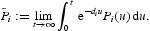

Clearly, *P˜*_*i*_ are the average time that recovered individuals remain in the recovered class before relapsing. By the properties of *P*_*i*_, one knows that

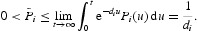

Actually, 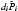 are the probability that recovered individuals will die during the recovery period. Hence, *Q*_*i*_ represent the proportion of the recovered individuals who could survive the recovery period, where

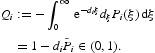

Define

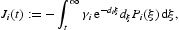

then *J*_*i*_(*t*)≥0, ∀ *t*>0, and 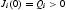.

The basic reproduction number 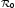 is defined as the expected number of secondary cases produced in an entirely susceptible population by a typical infected individual during their entire infectious period [8,9]. For system (5), we can calculate it as the spectral radius of a matrix called the next generation matrix. Let

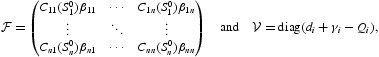

then the next generation matrix is

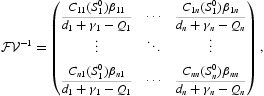

and hence the basic reproduction number of model (5) is calculated by the spectral radius of the next generation matrix

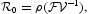

where ρ(·) denotes the spectral radius of matrix. It is well known that 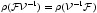. Thus

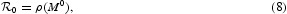

where

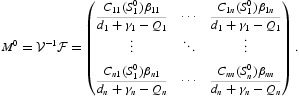

Since it can be verified that system (5) satisfies conditions 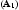
–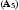 of [9, Theorem 2], we have the following proposition.

Proposition 1 For system (5), the DFE *P*_0_ is locally asymptotically stable if 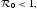 while it is unstable if 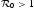.

Note that Equation (5) may not have an EE for finite time *t*. It follows from [30] that if Equation (5) has an EE, then the EE must satisfy the limiting system given by

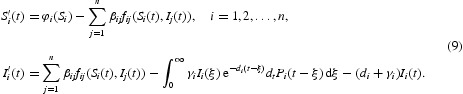

Since the limiting system (9) contains an infinite delay, its associated initial condition needs to be restricted in an appropriate fading memory space. For any 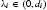, define the following Banach space of fading memory type (see e.g. [15,17] and references therein)

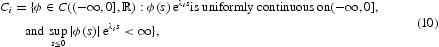

with norm 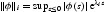. Let φ∈*C*_*i*_ be such that 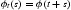, 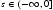. Let 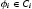 and 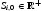 such that 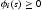, 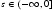. It turns to consider solutions of system (9), 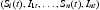, with initial conditions

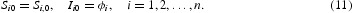

Standard theory of functional differential equations [17] implies *I*_*it*_∈*C*_*i*_ for *t*>0. Thus, we will consider system (9) in the phase space

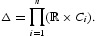

It can be verified that solutions of Equation (9) in Δ with initial conditions (11) remain non-negative.

An equilibrium 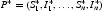 in the interior of Γ_0_ is called an EE, where 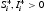 satisfy the equilibrium equations

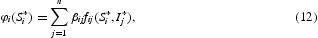

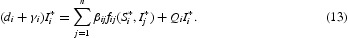

## 3. Global stability results

The global dynamical behaviour of system (5) and (9) is completely established in the following results. Denote

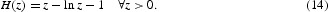

Obviously, 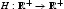 attains its strict global minimum at *z*=1 and *H*(1)=0.

### 3.1. Global dynamics of DFE

Theorem 3.1 Assume that the functions *f*_*ij*_ satisfy 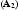
–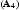, and the matrix 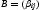 is irreducible. The following results hold for system (5) with 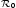 given in Equation (8).

(i) If 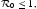 then the DFE of system (5) is globally asymptotically stable in Γ and there does not exist any EE.
(ii) If 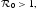 then the DFE is unstable and system (5) is uniformly persistent in Γ.

*Proof* Let us first define matrix value function

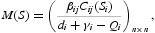

where 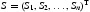. Note that *M*(*S*_0_)=*M* ^0^. Since 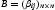 is irreducible, the matrix *M* ^0^ is also irreducible.

We first claim that there does not exist any EE P* in Γ. If we assume that *S*≠*S*_0_, it follows that 0<*M*(*S*)<*M* ^0^. Since non-negative matrix *M*(*S*)+*M* ^0^ is irreducible, it follows from the Perron–Frobenius theorem [4, Corollary 2.1.5] that 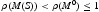. This implies that equation *M*(*S*)*I*=*I* has only the trivial solution *I*=0, where 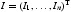. Hence the claim is true. Next we claim that the DFE *P*_0_ is globally asymptotically stable in Γ. From the Perron–Frobenius theorem [4, Theorem 2.1.4], we have that the non-negative irreducible matrix *M* ^0^ has a strictly positive left eigenvector 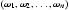 associated with the eigenvalue ρ(*M* ^0^) such that

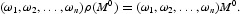

Let

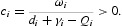

Consider a Lyapunov functional

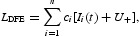

where *U*_+_ is given as 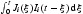. It is easy to know that 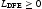 with equality if and only if *I*_*i*_(*t*)=0 and 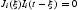 for almost all ξ≥0. Differentiating *U*_+_ along the solutions of system (5) and using integration by parts, we obtain

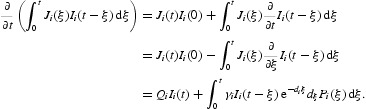

Thus, the derivative along the trajectories of system (5) is

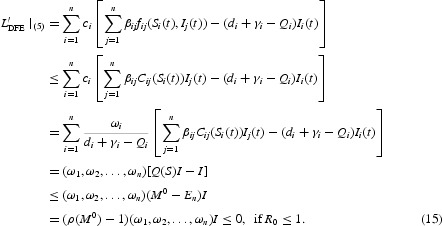

Where *E*_*n*_ denote the *n*×*n* identity matrix, 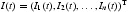. Let

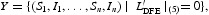

and *Z* be the largest compact invariant set in *Y*. We will show 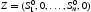. From inequality (15) and *c*_*i*_>0, 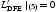 if and only if *I*=0. Then, by irreducibility of *B*, for each *j*, there exists *i*≠*j* such that 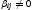, thus *I*_*j*_(*t*)=0, *j*=1, 2, … , *n*. Therefore, 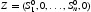, which implies that the compact invariant subset of the set where 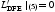 is only the singleton *P*_0_. Using Lemma 2.1 of [34] and the LaSalle–Lyapunov theorem (see [21, Theorem 3.4.7] or [15, Theorem 5.3.1]), we conclude that 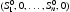 globally attracts all the solutions of model (5) if *R*_0_≤1. If *R*_0_>1 and *I*(*t*)≠0, it follows that

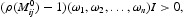

which implies that, in a sufficiently small enough neighbourhood of 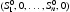, 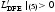. Therefore, 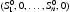 is unstable when *R*_0_>1. Using a uniform persistence result from [26,36] and a similar argument as in [27] and the proof of Theorem 3.2 of [34], we can show that the instability of *P*_0_ implies the uniform persistence of system (5) when *R*_0_>1. This completes the proof of Theorem 3.1.

Next we show that the EE P* of system (9) is unique and globally asymptotically stable when 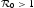. Summarizing the statements, uniform persistence of Equation (9) from Theorem 3.1, together with uniform boundedness of solutions in the interior of Γ, implies system (9) admits at least one EE [5, Theorem 2.8.6].

Let 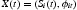 be a solution of Equation (9). By Theorem 3.1 and using similar arguments to [27], it follows that the ω-limit set *W* of *X* is non-empty, compact and invariant and that *W* is the union of orbits of Equation (9).

### 3.2. Global dynamics of EE

To get the global stability of P*, we make the following assumptions [31]:

(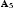): 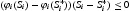, for *S*_*i*_≥0.
(**A**_6_) For 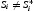, 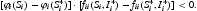

(**A**_7_) For *S*_*i*_, *I*_*j*_>0,

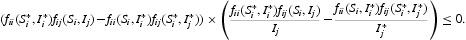

For convenience of notations, set

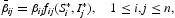

and

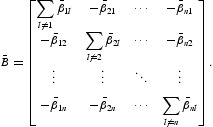

Then, 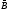 is also irreducible. One knows that the solution space of the linear system

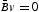

has dimension 1 and

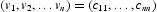

gives a base of this space where 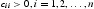, is the co-factor of the *i*th diagonal entry of 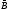.

Theorem 3.2 Consider system (9). Suppose that 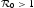 and functions *f*_*ij*_ and ϕ_*i*_ satisfy 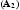
–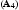 and 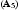
–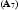, 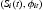 is a solution to Equation (9) that lies in Γ_0_, then

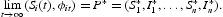

*Proof* Let 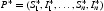 denote the unique EE of system (9). Define a Lyapunov functional as

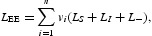

where

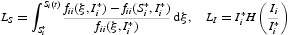

and

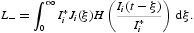

The definition of the fading memory space implies that *L*_EE_ is well-defined, that is, *L*_EE_ is bounded for all *t*≥0. It follows from Lemma 2.1 of [34] and assumptions (**A**_5_)–(**A**_6_) that 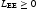 with equality if and only if 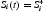, 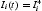 and 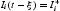 for almost all ξ≥0.

Differentiating *L*_*S*_ along the solution of system (9) and using equilibrium equations (12) and (13), we obtain

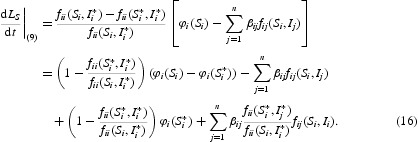

Differentiating *L*_*I*_ along the solution of system (9), we obtain

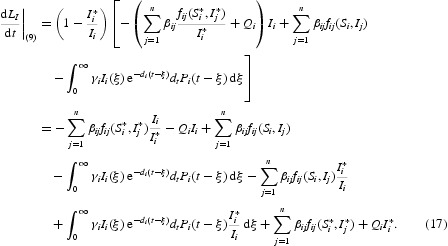

Differentiating *L*_−_ along the solution of system (9) and using integration by parts, we obtain

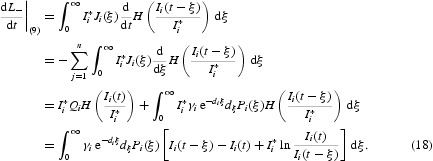

Combining Equations (16)–(18) yields

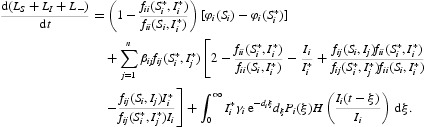

Let 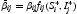 and

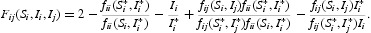

Then, by assumption 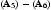,

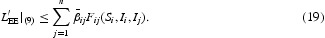

Furthermore, under 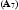, we have

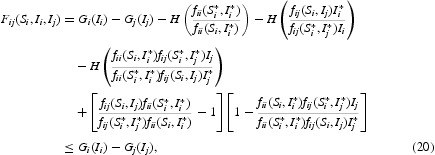

where 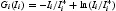 Obviously, the equalities in Equations (19) and (20) hold if and only if

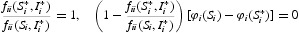

and

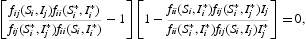

i.e. 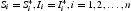. We can show that *F*_*ij*_ and 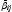 satisfy the assumptions of Theorem 3.1 and Corollary 3.3 in [22]. Therefore, the function 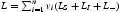 is a Lyapunov function for system (9), namely, 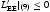 for all 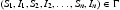. One can only show that the largest invariant subset where 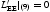 is the singleton P* using the same argument as in [22]. By LaSalle's invariance principle, P* is globally asymptotically stable in Γ_0_. This completes the proof of Theorem 3.2.

## 4. Special case for *n*=1

When *n*=1, system (5) reduces to a single-group SIR model with general relapse distribution and nonlinear incidence rate:

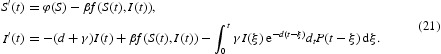

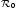
 will reduce to 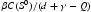, where 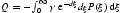. Following the method of constructing Lyapunov functionals,

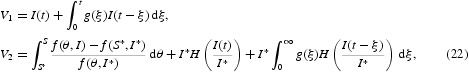

where *H* is given in Equation (14), and

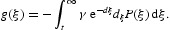

One can determine the global dynamics of single-group SIR model (21). Applying Theorems 3.1 and 3.2, we obtain the following global dynamics but omit the proof.

Theorem 4.1 Under the simpler version of assumptions 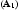
–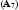 on functions ϕ and *f*, let (*S*(*t*), *I*(*t*)) be a solution to Equation (21). If *R*_0_>1, then 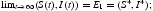 if *R*_0_≤1, then 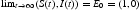.

*Remark 1* The results in Theorem 4.1 improve the corresponding results in [26], which gives part of the proof for this problem when 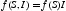 in system (9).

## 5. Numerical simulation

In this section, we carry out numerical simulation to illustrate and support our analytical results. We consider a simpler case in which all groups share the same natural death rate: *d*_*i*_=*d* for *i*=1, 2, … , *n*. Further, we denote 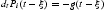 and assume that the functions *g*_*i*_(ξ) are disease-specific only, implying that 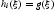 for *i*=1, 2, … , *n*. We choose the gamma distribution:

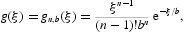

where *b*>0 is a real number and *n*>1 is an integer. This is widely used and can approximate several frequently used distributions. For example, when *b*→0^+^, *h*_*n, b*_(*s*) will approach the Dirac delta function, and when *n*=1, *h*_*n, b*_(*s*) is an exponentially decaying function. Following the technique and method in [35], define

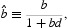

which can absorb the exponential term 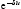 into the delay kernel. The second equation in Equation (5) can be rewritten as

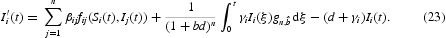

For *l*=1, … , *n*, let

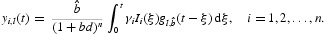

Thus, for 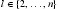, we obtain

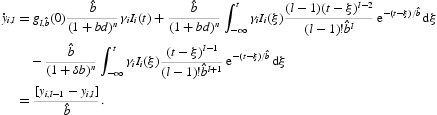

For *l*=1, we have

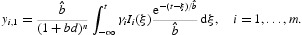

It follows that

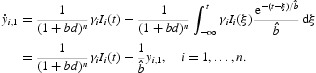

Thus, the integro-differential system (5) is equivalent to the ordinary differential equations

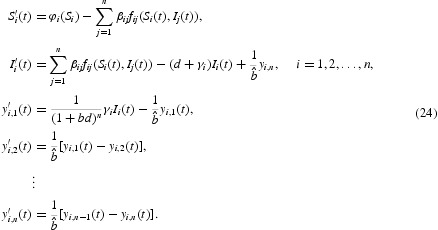

Consider the system (24) for the case

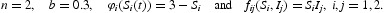

One then has a two-group model as follows:

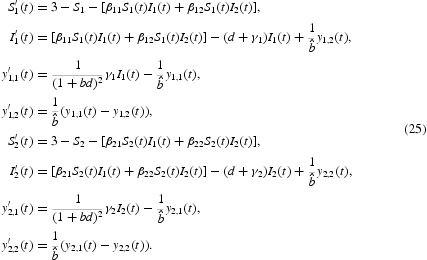

**Fig. 2.**
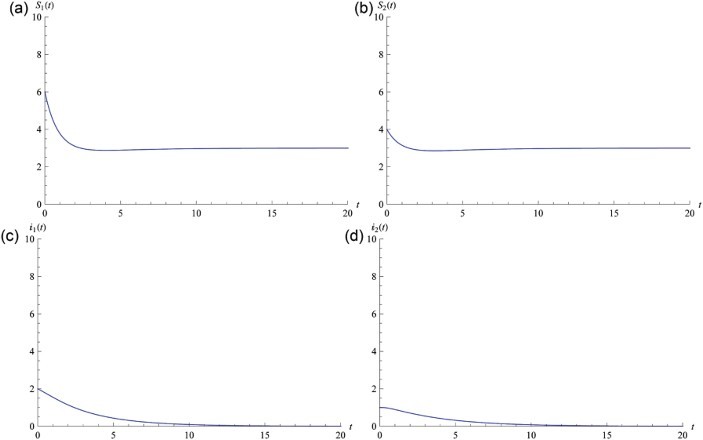
Numerical simulation of Equation (25) with *R*_0_=0.144355<1, hence *P*_0_=(3, 0, 0, 0, 3, 0, 0, 0) is globally stable. Graphs (a) and (b) illustrate that *S*_1_(*t*) and *S*_2_(*t*) will eventually lead to steady state. Graphs (c) and (d) illustrate that *I*_1_(*t*) and *I*_2_(*t*) will eventually lead to zero. Initial value is *S*_1_(0)=6, *S*_2_(0)=4, *y*_1, 1_(0)=0.1, *y*_1, 2_(0)=0.1, *y*_2, 1_(0)=0.1, *y*_2, 2_(0)=0.1, *I*_1_(0)=2, *I*_2_(0)=1.

**Fig. 3.**
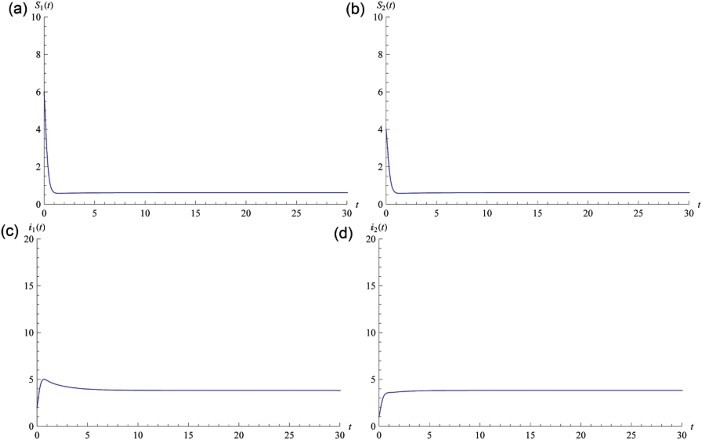
Numerical simulation of Equation (25) with *R*_0_=1.13422>1, hence P*=(0.621928, 3.82371, 0.377123, 0.377123, 0.621928, 3.82371, 0.377123, 0.377123) is globally stable. Graphs (a) and (b) illustrate that *S*_1_(*t*) and *S*_2_(*t*) will eventually lead to steady state. Graphs (c) and (d) illustrate that *I*_1_(*t*) and *I*_2_(*t*) will eventually lead to steady state. Initial value is *S*_1_(0)=6, *S*_2_(0)=4, *y*_1, 1_(0)=0.1, *y*_1, 2_(0)=0.1, *y*_2, 1_(0)=0.1, *y*_2, 2_(0)=0.1, *I*_1_(0)=2, *I*_2_(0)=1.

We adopt the following values for the parameters

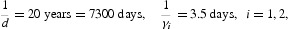

taken from the model parameters for herpes simplex virus type 2 presented by Blower *et al.* [6] and van den Driessche and Zou [10]. Moreover, we choose parameters from [6] as 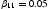, 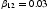, 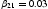 and 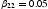. We can compute 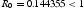, hence *P*_0_=(3, 0, 0, 0, 3, 0, 0, 0) is the unique equilibrium of system (25) and it is globally stable from Theorem 3.1 (Figure 2). For Theorem 3.2, we take the following parameters from [6,10]: 1/*d*=20, 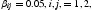 and 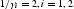. We can compute 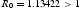, hence

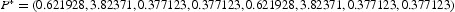

is the unique equilibrium of system (25) and is globally stable from Theorem 3.2 (Figure 3).

## 6. Summary and discussion

We present a complete mathematical analysis for global asymptotic stability of unique equilibrium P* of system (9); this complements our earlier work [34], where we assumed 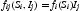.

Theorems 3.1 and 3.2 are also applicable to system (5) with other special nonlinear incidence rates appearing in the literature including 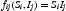 [12,13,19]; 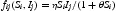 [1,11]; and 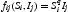 [24,25,37].

The new model includes many existing ones as special cases. For *n*=1, system (5) reduces to a single-group SIR model with general relapse distribution and nonlinear incidence rate. Biologically, Theorems 3.1 and 3.2 imply that, if the basic reproduction number 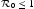, then the disease always dies out from all groups; if 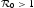, then the disease always persists in all groups at the unique EE level, irrespective of the initial conditions. On the other hand, Theorems 3.1, 3.2 and 4.1 demonstrate that heterogeneity and nonlinear incidence rate do not alter the dynamical behaviour of the SIR model with general relapse distribution and nonlinear incidence rate. Compared to results in [13,14], the group structure in system (9) greatly increases the complexity exhibited in the derivatives of the Lyapunov functionals. The key to our analysis is a complete description of the patterns exhibited in the derivative of the Lyapunov functionals using graph theory. Our approach may provide a frame work for dynamics of sexually transmitted diseases with relapse distribution and multi-group structure. Heterogeneity in the host population can result from different behaviours (e.g. numbers of sexual partners for some sexually transmitted infections). The global dynamics exclude the existence of Hopf bifurcation leading to sustained oscillatory solutions.

We should point here that this work is motivated by Yuan *et al.* [35] and Sun *et al.* [31] where disease with latency spreading in a heterogeneous host population and nonlinear incidence of infection and nonlinear removal functions between compartments was considered. In the proof, we utilize a graph-theoretical approach to the method of global Lyapunov functions that is motivated by the works in [13,14,18,19,22,23,26,31,34].

## Funding

J. Wang is supported by National Natural Science Foundation of China (no. 11201128), the Science and Technology Research Project of the Department of Education of Heilongjiang Province (no. 12531495), the Natural Science Foundation of Heilongjiang Province (no. A201211), and the Science and Technology Innovation Team in Higher Education Institutions of Heilongjiang Province. X. Liu is supported by the National Natural Science Foundation of China (11271303).
